# 727 Novel silver based â€œsuitâ€#157; dressing technique for Steven Johnson Syndrome and other cutaneous exfoliative conditions

**DOI:** 10.1093/jbcr/irac012.281

**Published:** 2022-03-23

**Authors:** Jake Laun, Kimberly Brown Maynell, Loryn Taylor, Lancia Simmons, Amanda Krenitsky, David J Smith

**Affiliations:** University of South Florida, Riverview, Florida; Tampa General Regional Burn Center, Tampa, Florida; Tampa General Regional Burn Center, Tampa, Florida; Tampa General Regional Burn Center, Tampa, Florida; University of South Florida, Tampa, Florida; University of, Tampa, Florida

## Abstract

**Introduction:**

Exfoliative skin conditions such as Steven Johnson Syndrome (SJS)/toxic epidermal necrolysis (TEN) and other significant drug related reactions are complex medical conditions that provide a challenge to the burn surgeon, especially with regards to local wound care. Various modalities of wound care require frequent dressing changes; however, these changes put the patient through significant pain and potentially harmful experiences that could lead to worse skin exfoliation, scarring and pigmentation changes. As part of our burn unit, we have created a dressing utilizing silver impregnated nylon sheets that limits the amount of wound care performed and therefore the amount of potential exfoliative damage.

**Methods:**

We have employed this means of dressing in all our Steven Johnson patients with significant open or blistered areas. We performed a retrospective analysis looking at our patients who were admitted with Steven Johnson Syndrome/toxic epidermal necrolysis or other exfoliative skin disorder over the last 7 years. We had 52 patients who ranged from having 2-100% of skin involved with significant blistering or exposed areas. The suit is made specific to the patient as each area is measured and the silver sheets are formed to the patient and secured in place. The silver sheets are saturated with sterile water and rewet with saline every four hours and changed every three days.

**Results:**

By utilizing these silver-based dressings, we have limited the amount of dressing changes and concomitant pain for patients while also limiting skin infections to only 1 out of our 52 patients. For blisters on the face, a local antibiotic ointment was used; and once the skin lesions had healed, a moisturizing lotion was used.

**Conclusions:**

Steven Johnson Syndrome and other exfoliative skin conditions require significant wound care. By minimizing dressing changes, one can lessen the pain to patients and by utilizing dressings that are infused with silver, one can also potentially decrease the risk for infection as was seen in our patient population.

